# Cognitive function, social integration and mortality in a U.S. national cohort study of older adults

**DOI:** 10.1186/1471-2318-9-33

**Published:** 2009-07-28

**Authors:** Thomas O Obisesan, RF Gillum

**Affiliations:** 1 Department of Medicine, College of Medicine, Howard University, Washington, DC, USA

## Abstract

**Background:**

Prior research suggests an interaction between social networks and Alzheimer's disease pathology and cognitive function, all predictors of survival in the elderly. We test the hypotheses that both social integration and cognitive function are independently associated with subsequent mortality and there is an interaction between social integration and cognitive function as related to mortality in a national cohort of older persons.

**Methods:**

Data were analyzed from a longitudinal follow-up study of 5,908 American men and women aged 60 years and over examined in 1988–1994 followed an average 8.5 yr. Measurements at baseline included self-reported social integration, socio-demographics, health, body mass index, C-reactive protein and a short index of cognitive function (SICF).

**Results:**

Death during follow-up occurred in 2,431. In bivariate analyses indicators of greater social integration were associated with higher cognitive function. Among persons with SICF score of 17, 22% died compared to 54% of those with SICF score of 0–11 (p < 0.0001). After adjusting for confounding by baseline socio-demographics and health status, the hazards ratio (HR) (95% confidence limits) for low SICF score was 1.43 (1.13–1.80, p < 0.001). After controlling for health behaviors, blood pressure and body mass, C-reactive protein and social integration, the HR was 1.36 (1.06–1.76, p = 0.02). Further low compared to high social integration was also independently associated with increased risk of mortality: HR 1.24 (1.02–1.52, p = 0.02).

**Conclusion:**

In a cohort of older Americans, analyses demonstrated a higher risk of death independent of confounders among those with low cognitive function and low social integration with no significant interaction between them.

## Background

Both impaired cognitive function and social isolation are prevalent concomitants of aging in industrialized nations [1,2]. Cognitive function has been found to be inversely associated with subsequent mortality in elderly adults in a number of previous studies [3-6]. Mechanisms remain obscure but may include diminished adherence to medical regimens, and self care including healthful diet and exercise. Likewise, social integration (close social relationships and ties to community) has been found to be inversely related to mortality [7-9]. Further, low social integration may be a risk factor for cognitive decline and dementia [2]. Mechanisms may include support received or provided, improved coping with stressful life events, reduced depression, and positive emotions leading to health-promoting physiological effects of decreased chronic sympatho-adrenal activation, improved immune function, and less chronic inflammation [10-13]. Greater social integration was associated with lower levels of C-reactive protein (CRP) [13]. One study suggests an interaction between social networks and Alzheimer's disease pathology, such that even at more severe levels of disease, individuals with larger network sizes had higher cognitive function [14]. However, further studies of interaction versus independence of cognitive function and social integration in prediction of mortality are needed to document such an important finding in the literature.

Therefore, we tested hypotheses of independent, inverse associations of score on a test of cognitive function and a social integration index with mortality in the United States population. We further test the hypothesis that the effect of cognitive function score on mortality is modified by social integration index, the effect being less in the well integrated than in the less well integrated. We report the analysis of data on mortality in a national health examination and follow-up survey conducted with scientific sampling and state-of-the-art interviewing, examination and laboratory methods.

## Methods

### Subjects

The Third National Health and Nutrition Examination Survey (NHANES III) was conducted in 1988–1994 on a nationwide multi-stage probability sample of 39,695 persons from the civilian, non-institutionalized population aged 2 months and over of the United States. Details of the plan, sampling, operation, response and institutional review board approval have been published as have procedures used to obtain informed consent and to maintain confidentiality of information obtained [15]. The personal interviews, physical and laboratory examinations of NHANES III subjects provided the baseline data for the study. This analysis was based on follow-up data collection through 2000. Of 33,994 persons with baseline interview data, 13,944 were under age 17 and 26 lacked data for matching leaving 20,024 eligible for mortality follow-up. Two deaths were excluded for missing data on cause of death. The NHANES III Linked Mortality File contains information based upon the results from a probabilistic match between NHANES III and the National Death Index records. The NHANES III Linked Mortality File provides mortality follow-up data from the date of NHANES III survey participation (1988–1994) through December 31, 2000.

Of the 20,022 interviewed persons with mortality follow-up, 6,588 were aged 60 years and over and eligible to have cognitive function testing performed, 6,339 of whom had valid cognitive function data. After excluding persons with missing data for marital status, education, self-reported health status, cigarette smoking status, history of stroke, history of heart attack, history of cancer (other than skin), social integration and mean systolic blood pressure at home visit, 5,908 persons aged 60 and over with complete data remained for this mortality analysis. The length of follow-up of survivors ranged from 75 to 146 months, mean 108 months.

#### Cognitive Function and Social Integration

During a home interview, an interviewer collected the socio-demographic variables such as age, gender and level of education used in this analysis. Questions assessing cognition were asked of respondents aged 60 or older and not to proxy respondents. These questionnaires were designed for administration in a bilingual (English/Spanish) format so that respondents could be interviewed in their preferred language. The neuropsychological measures used in the NHANES III study were selected to assess cognitive functions typically affected in dementia. The cognitive items on NHANES III are subsets of different cognitive screening instruments. For example, naming 3 objects comes from the Mini Mental Status Examination and subtracting 3 from 20 comes from the Short Portable Mental Status Questionnaire[16]. The orientation questions are common to most cognitive screens. These items were administered both at home interview and again at a mobile examination center to assess orientation, recall and attention [17,18]. To minimize non-response in older persons, a home examination consisting of abbreviated set of measures similar to those performed, was administered to 493 (8.6 percent of the sample for this analysis) participants who were unable or unwilling to come to a mobile examination center for a complete examination. Both examinations assessed memory function using the SICF. The version of SICF used consisted of six orientation, six recall and five attention items. The six orientation items include general information such as the day of the week, the date, and participant's complete address including street, city/town, state and zip code[17]. Each correct reply was scored one, with zero for an incorrect reply. Six recall items were tested in the home by naming three objects to the participant "apple", "table" and "penny", all of which were repeated immediately up to maximum of six trials and number of trials required to learn the task are noted. Each correct response was scored as one or scored zero for an incorrect answer irrespective of the number of trials required to learn the objects. The subjects were asked to recall after 2 minutes of distracting tasks. Again, each object recalled correctly was scored one and zero for an incorrect answer. Attention was evaluated in the home by asking the participant to count backwards by 3's from 20 each time. The series of digits were selected from those used in the Weschler Adult Intelligence Scale[19] Each correct digit was scored as one for correct count or zero for wrong count. Thus, the overall scores on SICF calculated from the replies use the sum of orientation, recall and attention and ranged from 0 to 17 with median of 13, 25^th ^percentile 12 and 75^th ^percentile 16, i.e. skewed to the left. For analysis, four groups were formed using these cut-points; quartiles with equal numbers of subjects could not be formed due to the skewed distribution and discrete nature of the variable. Two subscales (orientation/recall, range 0–12; counting, range 0–5) were also formed. Cronbach's apha for SICF was 0.77.

Social integration was measured using a social network index (SNI) as described in a previous analysis of these data [13]. Briefly, variables for marital status (1 married, 0 other), frequency of contacts in domains of friends and relatives (1 > = 156 0 < 156 contacts/year), religious attendance (1 > = 4/year, 0 <4/year) and voluntary associations (1 any memberships, 0 other) were summed and the resulting total ranging from 0 to 4 used in the analysis, as previously described.

#### Confounding and mediating variables

A review of the literature identified potential confounding variables: age, gender, race/ethnicity, education, and health status at baseline. Health status was assessed as self-reported general health, presence or absence of any history of major morbidity by physician diagnosis (heart attack, heart failure, stroke, medication for hypertension, diabetes, chronic bronchitis, emphysema, or non-skin cancer) and limitation of mobility (self-reported difficulty in climbing one flight of stairs or walking 1/4 mile with survey physician impression of mobility used to impute missing data). Possible mediators of the effect of social integration were leisure-time physical activity, regular clinic or physician, smoking, alcohol use, body mass index, blood pressure and C-reactive protein. Measurement of blood pressure, height, weight and serum analytes is described elsewhere [15].

#### Outcome Variable

NCHS conducted a mortality linkage of the Third National Health and Nutrition Examination Survey (NHANES III) with the National Death Index. The current linkage of the NHANES III includes deaths for adult participants occurring from the date of NHANES III interview through December 31, 2000. Information regarding the date of death and age of death, was collected from matched death certificates. Variables used in the selection step of the matching process were social security number, components of name and date of birth. This process detected 2,431 deaths in those in the present analysis. Efforts to trace all NHANES III participants who died may have been unsuccessful in some cases. However, previous validation studies of tracing in the NHANES I Epidemiologic Follow-up Study showed that only about one percent of deaths were not successfully identified using these methods [20]. For details about NHANES III Linked Mortality Files see .

### Statistical Analysis

Detailed descriptive statistics and measures of association were computed using the SUDAAN system (Version 9.0, Research Triangle Institute, Research Triangle Park, NC), to take into account the complex survey design and design effect in producing point and variance estimates using Taylor series linearization for variance estimation [21]. Kaplan-Meier survival curves were computed using PROC KAPMEIER. Estimates of the risk of death for persons with lower SICF score relative to those scoring 17 derive from Cox proportional hazards regression models with time to event as the time scale computed using the SURVIVAL procedure in SUDAAN. Follow-up time for survivors ended (was "censored") at the date of the last follow-up interview. An interaction term was initially included for social integration score with SICF score. Two models were fit: one controlled for likely confounders only and the second for confounders and likely mediators of the association of social integration with mortality. Validity of the proportional hazards assumption was confirmed by inspection of unweighted log negative log survival curves [22].

## Results

The mean SICF score for persons aged 60 years and over in 1988–1994 was 13.5, range 0–17, median 13, inter-quartile range (IQR) 12–16. The distribution was skewed to the left. Table 1 shows age-adjusted prevalence of selected characteristics by SICF category. SICF score was significantly associated with age, ethnicity, region, marital status, education, self-reported health status, mobility limitation, smoking, alcohol use, physical activity, regular source of care, systolic blood pressure and body mass index.

**Table 1:** Prevalence (%) of selected socio-demographic characteristics in persons aged 60+y by level of cognitive function: NHANES III.

		SICF score				
	Total	0–11	12–13	14–16	17	P*

N	5908	1867	1985	1177	988	

Total	100	20	36	20	23	

Female	100	22	36	20	22	0.15

Male	100	19	36	21	24	

Age 80+ y	100	37	27	24	12	0.00

70–79 y	100	22	35	20	24	

60–69 y	100	15	39	20	26	

Mexican American	100	37	25	26	12	0.00

Non-Mexican American	100	20	36	20	23	

African American	100	41	27	17	15	0.00

Non-African American	100	19	37	21	24	

South region	100	29	35	18	18	0.00

Other regions	100	17	36	21	25	

Metropolitan residence	100	17	40	20	22	0.02

Non-metro residence	100	23	33	21	24	

Unmarried	100	27	33	21	19	0.00

Married	100	16	38	20	26	

Educ < 12 y	100	32	32	19	19	0.00

Educ > = 12 y	100	12	39	20	29	

• *Chi-square test

**Figure 1:**
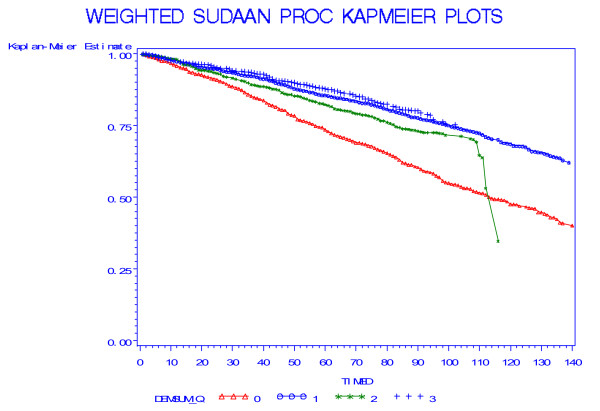
**Weighted Kaplan-Meier plots of survival over the follow-up period by level of the score on the short cognitive function index (triangles, 0–11; circles 12–13; stars, 14–16; plusses, 17)**.

**Figure 2:**
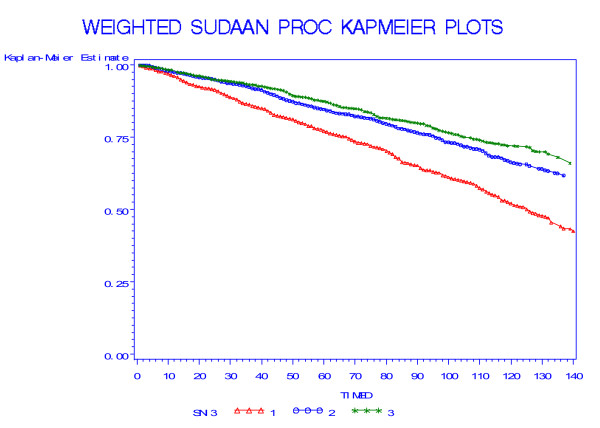
**Weighted Kaplan-Meier plots of survival over the follow-up period by level of the score on the Social Network Index (triangles, 0–1; circles 2; stars, 3–4)**.

Table 2 shows the percentage dying over the follow-up period by SICF score at baseline. (Due to rounding percentages may not total exactly 100). Compared to the survivors, scores of decedents were clustered in the two lowest categories (p < 0.0001). Kaplan Meier survival curves for persons aged 60 and over at baseline showed the poorest survival in those scoring 0–11 and the best survival in those scoring 17 (Figure 1). (The curve for the third group – line with stars – became unstable after 110 months due to the small number of events.) Similarly, Kaplan Meier survival curves for persons aged 60 and over at baseline showed the poorest survival in those in the lowest social integration index category and the best survival in those in the highest category (Figure 2).

**Table 2:** Prevalence (%) of selected biomedical characteristics in persons aged 60+y by level of cognitive function: NHANES III.

		SICF score				
	Total	0–11	12–13	14–16	17	P*

N	5908	1867	1985	1177	988	

Total	100	20	36	20	23	

Fair-poor health	100	30	32	19	19	0.00

Good health	100	16	38	21	25	

> = 1 chronic illness	100	21	35	21	23	0.21

No chronic illness	100	19	37	20	24	

Mobility limitation	100	30	38	17	15	0.00

No mobility limitation	100	16	35	22	27	

Current smoking	100	22	36	17	24	0.02

No smoking	100	17	38	21	25	

Alcohol in past month	100	13	40	17	30	0.00

No alcohol	100	24	34	22	20	

Low physical activity	100	26	34	20	21	0.00

Mod/hi activity	100	21	35	20	24	

No regular physician	100	23	39	19	19	0.08

Regular physician	100	20	35	21	24	

No religious attendance	100	22	37	20	21	0.22

Wkly relig attendance	100	19	36	20	24	

Low social support	100	27	36	20	17	0.01

High social support	100	19	36	21	24	

Systolic BP > = 140 mmHg	100	19	38	19	24	0.01

Systolic BP < 140 mmHg	100	20	36	20	24	

BMI > = 25 kg/m2	100	18	35	22	25	0.03

BMI < 25 kg/m2	100	23	37	18	22	

Death during follow-up	100	32	35	17	15	0.00

Alive during follow-up	100	14	36	22	27	

• *Chi-square test

Proportional hazards regression analysis revealed a significant bivariate inverse association of SICF score category with mortality during follow-up: test for trend hazard ratio (HR) 0.79, 95% CL 0.73–0.85, p < 0.001. Compared to persons scoring 17, those scoring 0–11 had a hazards ratio of 2.2 (95% CL 1.7–2.7, p < 0.001). Regression models failed to find significant hypothesized interaction between SICF score and social integration index (P = 0.10).

Controlling for confounding by baseline demographic and socioeconomic variables and health status (Model 1), persons scoring 0–11 on the SICF had significantly higher risk of mortality than those scoring 17 (Table 3). A test for linear trend was significant (p = 0.03). Next, the effect was assessed after controlling for variables identified in the literature as potential mediators of a protective health effect of social integration (e.g. healthy behaviors) and social integration index itself. Table 3 shows that these variables explained little of the effect of SICF score (Model II). Further, a significant protective effect of high social integration index independent of SICF score and other variables was apparent (p = 0.02). Finally, to assess whether the beneficial effect of high social integration might be mediated by reduced inflammation, we added the log concentration of C-reactive protein to Model II (not shown). This had essentially no effect on the hazard ratio for low social integration (HR = 1.23) nor on the hazard ratio for low cognitive function (HR = 1.35), indicating that neither effect could be explained by inflammation.

**Table 3:** Adjusted hazards ratios (95% confidence intervals) of SICF score for mortality from all causes among persons aged 60+ y in NHANES III

Variable		Model I		Model II	
		Hazard ratio	95% CI	Hazard ratio	95% CI

	17	1.00		1.00	
	
SICF	14–16	1.10	0.82–1.80	1.11	0.82–1.49
	
score	12–13	1.07	0.85–1.35	1.06	0.84–1.35
	
	0–11	1.43*	1.13–1.80	1.36+	1.06–1.76

Age	Yr	1.09*	1.08–1.10	1.09*	1.08–1.10

Gender	Male	1.57*	1.42–1.73	1.57*	1.39–1.78

Ethnicity	AA	1.01	0.88–1.17	1.07	0.91–1.26
	
	MA	0.72	0.58–0.91	0.81	0.59–1.12

Education	< HS	1.01	0.88–1.16	0.96	0.84–1.09

Region	South	0.94	0.76–1.15	0.88	0.70–1.12

Urbanized	Yes	1.05	0.87–1.26	1.02	0.83–1.25

SR health	F/P	1.72*	1.49–1.99	1.47*	1.27–1.71

Morbidity	Yes	1.55*	1.35–1.79	1.48*	1.28–1.72

Mobility	Limited	1.25*	1.08–1.45	1.29*	1.13–1.48

SBP	Mm Hg			1.00	1.00–1.00

BMI	Kg/m2			0.96*	0.94–0.97

Smoking	Current			1.69*	1.39–2.06
	
	Former			1.34*	1.14–1.58

Alcohol	Yes			0.79*	0.67–0.94

Physical activity	Low			1.75*	1.52–2.02
	
	Average			1.26*	1.10–1.44

Reg. care	Yes			1.05	0.87–1.25

Social-network index	3–4			1.00	
	
	2			1.02	0.88–1.19
	
	0–1			1.24+	1.02–1.52

CI, confidence interval

SR self-reported; F/P fair-poor; Reg. care, regular care by personal physician.

* p < = 0.01, +P < 0.05

## Discussion

NHANES III data show that risk of mortality was higher among persons with low compared to high cognitive function and low compared to high social integration even after controlling for confounding by baseline health status and mobility, and health behaviors, supporting the hypothesis of an independent association. The association did not differ by gender, ethnicity or age. Cognitive function and social integration did not significantly interact.

Mechanisms by which low cognitive function or dementia adversely affect mortality remain obscure, for in most studies the effect cannot be fully explained by adjusting for comorbidity, functional status, or socio-demographic variables [3-6,23,24]. Low social integration has been linked to increased risk of cognitive decline [2,25]. Biopsychosocial mechanisms by which those who are well integrated in their society would be less likely to die when they experience cognitive impairment might include improved coping with the stressful life events of aging leading to decreased oxidative stress and free radical production leading to a slowing of the rate of progression of atherosclerosis in response to impaired cognitive function induced inactivity and adherence to therapy.

Recent work has explored the relationship of inflammation and cognitive function indicating an inverse association of levels of interleukin-6 and CRP with incident cognitive impairment, with mixed results regarding rate of change in cognitive function [26]. Another study in a sample with a mean age of about 45 years found that log IL-6 was significantly inversely correlated with performance on the Wisconsin Card Sorting Test, the Stroop color/word Test, the California Verbal Learning Test, and positively with the Trail Making Test (A & B); also, IL-1 alpha concentration was significantly inversely correlated with the California Verbal Learning Test [27]. After controlling for demographic variables and pro-inflammatory cytokines (linear regression), higher log IL-6 predicted lower scores on the color/word condition of the Stroop Test and higher scores on Trail Making Test A. Additionally, after controlling for demographic variables, inflammatory illness status, and proinflammatory cytokine concentration (linear regression), Log IL-1-alpha predicted log perseverative errors on the Wisconsin Card Sorting Test. The directionality of the association is unclear. The present study suggests that excess mortality associated with low social integration or low cognitive function is not explained by inflammation as measured by CRP. However, CRP in NHANES III was not high sensitivity (hs-CRP) and may therefore not sensitive enough to detect a relationship with AD-related pathology.

Nor is there evidence that excess mortality is explained by adverse effects of cognitive dysfunction on social integration nor was there an interaction of the two variables. A small study of hospitalized or day patients with significant cognitive impairment found no significant association of living alone, support from relatives, day centre care or home help with survival [28]. Receiving meals on wheels was associated with poorer survival. Mechanisms by which social integration may lessen risk of mortality include material aid from others, and emotional support to lessen the psychoneuroimmunologic effects of life stress on the organism [10-13].

NHANES III provides population-based data on the association of cognitive function, social integration and survival in a nationwide, representative sample of Americans. However, several unavoidable limitations of the present study include possible bias arising from survey non-response and from missing values for some variables and from possible changes in cognitive function and social integration or other variables over the follow-up period. A comparison of persons excluded with those included indicated that those excluded were significantly more likely to be over 80 and African American, but did not differ in gender, region or urbanization. Several special studies of NHANES III data have indicated little bias due to non-response [29]. Comparison of vital status and demographics of the analysis sample and those excluded for missing data but eligible for follow-up revealed those excluded were more likely to be female, Mexican American, in poor health, and more likely to die during the follow-up. Thus, selection bias cannot be excluded. It seems reasonable to suggest that the score of our index of cognitive function measures cognitive functioning, but the possible problems inherent in using a cognitive functioning screen that has not been used before, has no validity studies and cannot be directly compared to accepted cognitive screens used in the literature must be acknowledged [16]. We have explored two subscales (orientiation/recall and counting) as shown in the Appendix. The index of social integration used originates from work by Berkman and Syme [7]. However, their Social Network Index differs somewhat from what is described in this paper due to limitations of data in NHANES [13]. Further, it does not capture giving or receiving support, shown to be important for health [10]. Different results may have been obtained had other tests of cognitive function or social integration been used. Unfortunately we were unable to control for depressive symptoms due to lack of such data for persons over 60 years in the survey. CRP is a nonspecific inflammatory marker, which may not be sensitive to chronic brain tissue damage. The representativeness of the sample and the use of sample weights provide generalizability of the results to United States non-institutionalized population of the same ages. Data from longitudinal studies with multiple measures of multiple dimensions of cognition and integration would be helpful in delineating mechanisms involved.

## Conclusion

In a nationwide cohort of Americans, analyses demonstrated a higher risk of death independent of confounders among those with low cognitive function or social integration compared to others. The two variables did not interact. Given the growing global burden of impaired cognitive function and social isolation among the elderly, further research is need on the mechanisms by which these factors influence mortality.

## Competing interests

The authors declare that they have no competing interests.

## Authors' contributions

RG participated in the conception, design, analysis and interpretation of the data. TO participated in the conception, design, analysis and interpretation of the data. All authors read and approved the final manuscript.

## Appendix

### Cognitive Function Subscale Analyses

The mean SICF orientation/recall score for persons aged 60 years and over in 1988–1994 was 8.7, range 0–12, median 8.0, inter-quartile range (IQR) 7–11. The distribution was skewed to the left. For analysis the score was categorized into quartiles. The mean SICF counting score for persons aged 60 years and over in 1988–1994 was 3.9, range 0–5, median 5, inter-quartile range (IQR) 1–5. Over 50% had a score of 5; therefore the score was dichotomized as 5 versus < 5. In unadjusted bivariate crosstabulation, mortality was 48% of those in the lowest quartile, 30% in the second, 31% in the third and 22% in the highest quartile of orientation/recall (p < 0.001) and mortality was 43% in those with counting score < 5 versus 29% in those scoring 5 (p < 0.001). In proportional hazards regression analysis.

There were no significant interactions of orientation/recall with age, gender or race/ethnicity. The baseline orientation/recall score was a significant predictor of future survival after adjusting for sociodemographic and health status variables (p = 0.03). Compared to the highest quartile, the hazard ratio for the bottom quartile of the score was 1.23 (95% CL 1.01–1.49, p = 0.04). Similarly, compared to those with counting score of 5, those with counting score of < 5 had an adjusted hazard ratio of 1.22 (95% CL 1.05–1.41, p = 0.01). Hence both subscales of the SICF were significantly associated with survival.

## Disclaimer

The findings and conclusions in this report are those of the authors and do not necessarily represent the views of Howard University or the funding agency.

## Pre-publication history

The pre-publication history for this paper can be accessed here:


